# Classical celiac disease is more frequent with a double dose of HLA-DQB1*02: A systematic review with meta-analysis

**DOI:** 10.1371/journal.pone.0212329

**Published:** 2019-02-14

**Authors:** Judit Bajor, Zsolt Szakács, Nelli Farkas, Péter Hegyi, Anita Illés, Margit Solymár, Erika Pétervári, Márta Balaskó, Gabriella Pár, Patrícia Sarlós, Ákos Szűcs, József Czimmer, Kata Szemes, Orsolya Huszár, Péter Varjú, Áron Vincze

**Affiliations:** 1 Division of Gastroenterology, First Department of Medicine, University of Pécs, Medical School, Pécs, Hungary; 2 Clinical Medicine Doctoral School, University of Szeged, Szeged, Hungary; 3 Institute for Translational Medicine, University of Pécs, Medical School, Pécs, Hungary; 4 Institute of Bioanalysis, University of Pécs, Medical School, Pécs, Hungary; 5 Hungarian Academy of Sciences-University of Szeged, Momentum Gastroenterology Multidisciplinary Research Group, Szeged, Hungary; 6 First Department of Surgery, Semmelweis University, Budapest, Hungary; University Hospital Llandough, UNITED KINGDOM

## Abstract

**Background and aims:**

Experimental data suggest that the HLA-DQ2 gene dose has a strong quantitative effect on clinical outcomes and severity of celiac disease (CD). We aimed to conduct a meta-analysis with systematic review to investigate the association between HLA-DQB1*02 gene doses and the characteristics of CD.

**Methods:**

We searched seven medical databases for studies discussing HLA-DQB1 gene dose in CD and various disease characteristics, such as clinical presentation, histology, age at diagnosis, and comorbidities. Odds ratios (OR, for categorical variables) and weighted mean differences (for age) were calculated to compare patients with a double dose of HLA-DQB1*02 versus those with single and zero doses. Heterogeneity was tested with I^2^-statistics and explored by study subgroups (children and adults).

**Results:**

Twenty-four publications were eligible for meta-analysis. Classical CD was more frequent with a double versus single dose of the HLA-DQB1*02 allele (OR = 1.758, 95%CI: 1.148–2.692, I^2^ = 0.0%). In pediatric studies, gene dose effect was more prominent (OR = 2.082, 95%CI: 1.189–3.646, I^2^ = 0.0% and OR = 3.139, 95%CI: 1.142–8.630, I^2^ = 0.0% for the comparisons of double versus single and double versus zero dose, respectively). Atrophic histology was more prevalent with a double versus zero dose (OR = 2.626, CI: 1.060–6.505, I^2^ = 21.3%). We observed no gene dose effect regarding diarrhea, age at diagnosis, the severity of villous atrophy, and the association with type 1 diabetes mellitus.

**Conclusion:**

A double dose of HLA-DQB1*02 gene seems to predispose patients to developing classical CD and villous atrophy. Risk stratification by HLA-DQB1*02 gene dose requires further clarification due to the limited available evidence.

## Introduction

Celiac disease (CD) is an immune-mediated systemic disorder triggered by gluten that occurs in genetically susceptible individuals [1, 2]. CD is characterized histologically by small intestinal mucosal damage, clinically by various intestinal and extraintestinal manifestations.

The presence of HLA-DQ2 or DQ8 is essential in the disease pathogenesis. T-lymphocytes recognize gliadin peptides presented by antigen presenting cells expressing DQ2 or DQ8 on cell surface, exclusively. Therefore, theoretically, either haplotype must be present in all CD-patients [3].

HLA-DQ2 is present in up to 90–95% of celiac cases. The HLA-DQ2 heterodimer consists of an α and a β subunit encoded by HLA-DQA1*05 and HLA-DQB1*02 alleles on chromosome 6, respectively [3]. Alleles are located on the same chromosome in *cis* configuration (DR3/DQ2 haplotype) or separately on homologous chromosomes in *trans* configuration (DR5/DQ7 and DR5/DQ2 haplotypes) [3]. The two types of DQ2 heterodimers are DQ2.5 (DQA1*0501/B1*0201) and DQ2.2 (DQA1*0201/B1*0202). Patients with heterodimers of DQ2.5 carry a high risk and with heterodimers of DQ2.2 carry a low risk of CD [2, 4, 5]. DQ2.2 molecules are structurally similar to DQ2.5, but the latter’s gluten peptide-binding properties are less prominent [3, 4, 6]. Those with DQ2.2 haplotype are at high risk of CD but only if they are DQ2.2/2.5 or DQ2.2/DQ7 heterozygotes. In the latter case, functional DQ2.5 molecules can be assembled from α and β chains encoded separately on different chromosomes (DQA1*0505 and DQB1*0202, respectively); this constitution is called ‘DQ2 in trans’ [2, 5, 7, 8]. HLA-DQ8 is found up to 5–10% of CD patients, whose α and β chains are encoded by HLA-DQA1*0301 and HLA-DQB1*0302, respectively (that is, DR4/DQ8 haplotype with DR4-linked inheritance). A small minority of patients have half of the DQ2 heterodimer (either DQA*05 or DQB*02); however, it seems to be sufficient for effective antigen presentation [2, 5, 8].

Since the DQ2 molecule plays a crucial role in CD pathogenesis, the number of HLA DQB1*0201 copies might have important consequences in CD patients: 4 αβ-chain combinations can be synthesized in heterozygotes but all HLA-DQ molecules are identical in homozygotes [5, 9, 10]. Experimental data support this connection: HLA-DQ2.5 homozygotes can present gluten peptides on antigen-presenting cells more effectively than HLA-DQ2.5 heterozygotes [4, 5]. HLA-DQ2.5 homozygotes are at fivefold risk of CD as compared to HLA-DQ2.5 heterozygotes [6, 9–11]. The presence of a second β chain seems decisive in determining the risk, whereas the role of a second α chain appears less important [10, 12, 13]. The magnitude of immune response depends on gene dose: HLA-DQ2.5 homozygotes show maximal T-cell activation and proinflammatory response, whereas heterozygotes exhibit less prominent responses. The more DQ2.5 molecules are expressed on antigen presenting cells, the stronger the immune activation is [4–6]. Based on these *in vitro* immunological studies, one might assume that HLA-DQ2 homozygosity alters the course of CD; furthermore, the gene dose effect is manifested *clinically* with an increased risk of complications [14].

To our best knowledge, no meta-analyses have been conducted on the clinical effects of HLA-DQB1*02 gene dose in CD. It is assumed that patients with a double dose of HLA-DQB1*02 exhibit worse clinical outcomes, though some papers reported on how HLA-DQ2 gene dose (i.e., the number of DQB1*02 alleles) is associated with CD phenotype. This meta-analysis aims to investigate the association between HLA-DQB1*02 gene dose and the disease characteristics.

## Materials and methods

We conducted our meta-analysis observing the rules of the Preferred Reporting Items for Systematic Reviews and Meta-Analysis (PRISMA) Statement (S1 Appendix) [15].

### Search

PubMed (MEDLINE), Embase, Cochrane Controlled Register of Trials (CENTRAL), Web of Science, WHO Global Health Library, ClinicalTrials.gov, and Scopus were searched for articles discussing gene dose effect from inception until 22^nd^ October 2018. Our query was “celiac AND (homozyg* OR heterozyg* OR "gene dose" OR "gene dosage" OR "double dose" OR "double dosage" OR "single dose" OR "single dosage" OR "zero dose" OR "zero dosage" OR DQB1 OR DQ2.5 OR DQ2.2 OR DQ7 OR DQ8 OR "DQ2 in trans")”. Reference lists and citing articles of the relevant studies were hand-searched for further papers. No filters were imposed upon the search. Draft of search in Embase is presented in S2 Appendix.

Our PICO format was, as follows: (P) celiac disease, (I_1_) zero-dose HLA-DQB1*02, (I_2_) single-dose HLA-DQB1*02, (C) double-dose HLA-DQB1*02, (O) celiac phenotype at diagnosis including histological severity (atrophic vs. non-atrophic and Marsh 3c vs. Marsh 3a-b), clinical presentation (classical vs. non-classical and diarrhea vs. non-diarrhea), age at onset and at diagnosis, celiac-specific serology; concomitant immune-mediated disorders, dermatitis herpetiformis, anemia, dental complications, and malignant tumors.

### Eligibility, selection, and data extraction

We included original papers and conference abstracts, with the exclusion of case reports; and excluded comments, letters, editorials, and review articles. Eligible study design included both observational and experimental studies with adequate description of the genetic background and disease characteristics.

Eligible studies discussed celiac patients diagnosed in accordance with the current guidelines with known gene dose of HLA-DQB1*02 (double, single, and zero doses) and reported at least one of our predefined outcomes of patients by genotypes, separately. On inclusion, only PCR-based HLA-typing (sequence-specific primer or oligonucleotide probes) was acceptable.

Outcomes included clinical presentation dichotomized into classical and non-classical phenotype according to the Oslo criteria. Classical and non-classical CD are defined by the presence and absence of signs and symptoms of malabsorption (i.e., diarrhea, steatorrhea, weight loss, or growth failure), respectively [16], or into groups with and without diarrhea. Age at diagnosis and at disease onset were assessed separately. On one hand, diagnostic histology was divided into atrophic (Marsh 3 grade) and non-atrophic (Marsh 0–2 grades) mucosal damage [17]. On the other hand, we analyzed the severity of villous atrophy graded by the Marsh-Oberhuber classification (Marsh 3c vs. Marsh 3a-b) [18]. Tissue transglutaminase antibodies and endomysial antibodies were in the focus when discussing diagnostic serology. Other outcomes included disease complications and comorbid conditions (i.e., anemia, osteoporosis, autoimmunity, dental complications, dermatitis herpetiformis, malignant tumors, and type 1 diabetes mellitus).

We combined the yield of search in a reference manager software (EndNote X7.4, Clarivate Analytics, Philadelphia, PA, US), followed by the removal of overlaps between database content and duplicate records. The duplicate-free pool was searched first by title, then by abstracts, and full-texts against our eligibility criteria. Each phase of selection was carried out by two independent review authors (PV and KS) in duplicate, discrepancies were resolved by third party (MS) arbitration. Investigators had no contact with the authors of the original papers.

The following data were collected by two investigators onto pre-constructed Excel sheets: publication data, study design, population (numbers and characteristics of participants), HLA-DQB1*02 gene dose, age at onset; age, histology, serology, clinical presentation, anemia at diagnosis; concomitant immune-mediated disorders and complications: type 1 diabetes mellitus, dermatitis herpetiformis, dental enamel defect, recurrent aphthous stomatitis, enteropathy-associated T-cell lymphoma (EATL), small bowel carcinoma (SBC).

In the case of articles giving only HLA-DQ genotype, HLA-DQB1*02 gene dose was calculated, as follows: double-dose—HLA-DQ2.5 homozygotes (DQ2.5/DQ2.5) and compound heterozygotes (DQ2.5/DQ2.2); single-dose—HLA-DQ2.5 heterozygotes (DQ2.5/DQX) and HLA-DQ2 in trans (DQ2.2/DQ7); and zero-dose—HLA-DQ8/DQX and HLA-DQ2.2/DQX, where X represents any alleles except for DQ2.5 [8, 19–28].

### Risk of bias assessment

Two authors (MB and EP), unblinded to publication data, assessed the methodological quality of each study by using a tool developed *a priori* by our review team based on the Newcastle-Ottawa Scale (S1 Table). Results of risk of bias assessment were taken into account when assessing the limitations of the individual studies.

### Statistical analysis

A biostatistician (NF) carried out the statistical analysis by using Comprehensive Meta-Analysis software (Version 3, Biostat, Englewood, NJ). The random effect model with DerSimonian-Laird estimation was used for analysis [29]. For dichotomous outcomes (i.e., histology, clinical presentation, and diarrhea), we calculated odds ratios (ORs) and 95% confidence interval (CIs). For age at diagnosis, weighted mean difference (MD) and 95% CIs were calculated. Statistical significance was attained when *p*<0.05.

Heterogeneity was tested with I^2^- and chi^2^-tests. An I^2^ of 0%-40%, 30%-60%, 50%-90%, and 75%-100% represented not important, moderate, substantial, and considerable between-study heterogeneity with *p*<0.10 indicating statistical significance [30]. Since the clinical phenotype of CD diagnosed in childhood and adulthood may differ, we planned to set up study subgroups by age (children and adults) in each plot [31–35]. The number of studies was insufficient for meta-regression by gene dose.

Funnel plots were used to assess publication bias.

Sensitivity analysis was performed by omitting studies one-by-one from the analyses and recalculating the pooled effect.

## Results

### Search and selection

**Fig 1:**
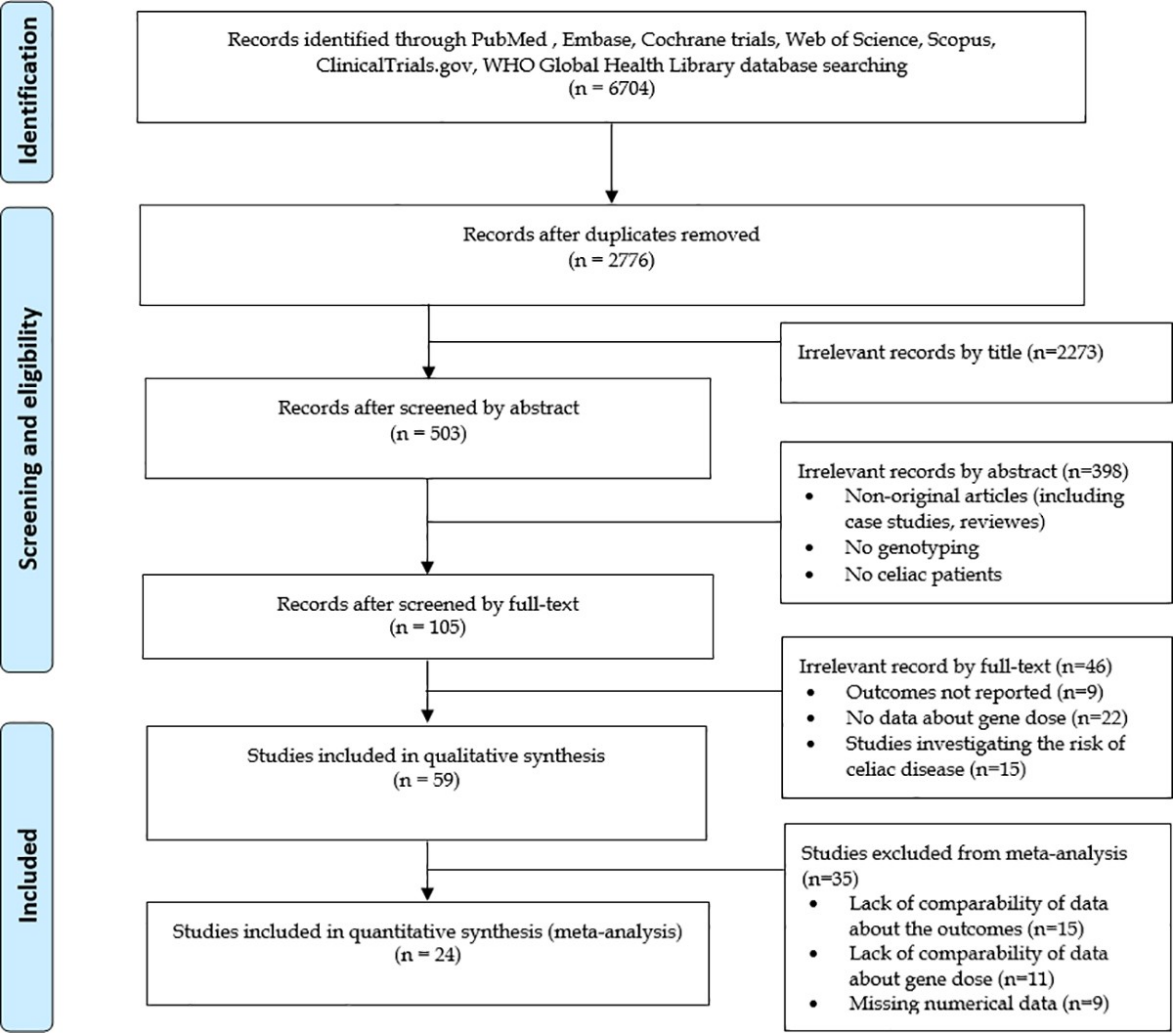
Flow chart of meta-analysis.

Fig 1. shows the flowchart of this work. Our search strategy yielded 6704 records (PubMed [MEDLINE]: 954, Embase: 2277, CENTRAL: 43, Web of Science: 925, WHO Global Health Library: 795, ClinicalTrials.gov: 6, and Scopus: 1704). Out of a total of 59 papers eligible for qualitative synthesis (Table 1 and S2 Table) [7, 8, 12–14, 19–28, 36–79], 24 were included in meta-analysis (Table 1) [8, 12, 19–28, 36–47]. Results of meta-analysis are summarized in Table 2, raw data on disease characteristics are presented in S3 Table.

**Table 1:** Characteristics of included studies.

Author (year)	Country	Settings	N^0^ of pts.	Age group	Genotyping		
					Method	Target of typing within the study	N^0^ of pts. (double/single/zero dose)
Akar et al. (2015) [36]	Turkey	prospective, single center, cross-sectional	36 CD pts	children	PCR-SSP	HLA-DQB1*0201 allele dose	5/24/7
Araya et al. (2015) [19]	Chile	prospective, single center, case-control	56 CD pts and 166 first degree relatives	children and adults	PCR-SSP	HLA-DQ genotype	12/25/7
Bastos et al. (2017) [20]	Brazil	prospective, multicenter, case-control	66 CD pts and 32 CD/T1DM pts	not reported (median age 14 years)	RT-PCR	HLA-DQ genotype	16/52/30
Cabrera et al. (2018) [21]	Spain	prospective, single center, case-control	196 CD pts and 206 healthy control	children	PCR-SSO	HLA-DQ genotype	79/103/14
Cakir et al. (2014) [22]	Turkey	prospective, single center, cross-sectional?	78 CD pts	children	PCR-SSO	HLA-DQ genotype	28/34/14
Colombe et al. (2015) [37]	USA	retrospective, single center, cross-sectional	89 CD pts	adults	PCR-SSP	HLA-DQB1*0201 allele dose	45/7/37
Congia et al. (1994) [38]	Italy	prospective, single center, case-control	62 CD pts and 89 healthy control	children and adults	PCR-SSO	HLA-DQB1*0201 allele dose	30/31/1
Eller et al. (2006) [39]	Israel	prospective, single center, cross-sectional	175 Beduin kindred	children and adults	PCR-SSO	HLA-DQB1*0201 allele dose	3/3/0
Greco et al. (1998) [40]	Italy	prospective, single center, cross-sectional	145 CD pts	children	PCR-SSO	HLA-DQB1*02 allele dose	46/84/15
Gudjonsdottir et al. (2009) [23]	Sweden, Norway	prospective, multi-center, cross-sectional	224 CD pts (HLA status was available: 98 pts)	children and adults	PCR-SSO	HLA-DQ genotype	40/52/6
Hanif et al. (2017) [24]	Pakistan	prospective, single center, observational	12 CD pts	children	PCR	HLA-DQ genotype	5/7/0
Jores et al. (2007) [12]	Italy	retrospective, single center, cross-sectional	187 CD pts	children	PCR-SSO	HLA-DQB1*0201 allele dose	77/93/17
Kabatova et al. (2017) [25]	Slovakia	retrospective, single center, cross-sectional	258 CD pts (HLA status was available: 217 pts)	children	PCR-SSP	HLA-DQ genotype	42/97/78
Karinen et al. (2006) [41]	Finland	prospective, single center, cross-sectional	144 CD pts (only siblings from 52 families)	adults	PCR-SSP	HLA-DQB1*0201 allele dose	32/103/9
Mohammed et al. (2014) [42]	Egypt	prospective, single center, case-control	31 CD/T1DM pts	children and adults	PCR-SSP	HLA-DQB1*02 allele dose	16/8/7
Nenna et al. (2008) [43]	Italy	prospective, single center, cross-sectional	124 CD pts	children	PCR-SSP	HLA-DQB1*02 allele dose	26/85/13
Ros et al. (2010) [26]	Spain	retrospective, single center, cross-sectional	396 CD pts	children	PCR-SSO	HLA-DQ genotype	168/206/17
Rostami-Nejad et al. (2014) [8]	Iran	retrospective, multicenter, case-control	59 CD pts and 151 healthy control	children and adults	PCR-SSP	HLA-DQ genotype	15/30/14
Schweiger et al. (2016) [27]	Slovenia	prospective, single center, case-control	68 CD pts vs 69 CD/T1DM pts	children	PCR-SSO and -SSP	HLA-DQ genotype	41/81/12
Thomas et al. (2009) [44]	UK	retrospective, single center, cross-sectional	384 CD pts (HLA status was available: 360 pts)	adults	PCR-SSP	HLA-DQB1*0201 allele dose	71/247/42
Vegas-Sanchez et al. (2015) [45]	Spain	retrospective, single center, cross-sectional	14 CD pts	adults	PCR-SSO	HLA-DQB1*0201 allele dose	2/7/3
Vermeulen et al. (2009) [28]	The Netherlands	retrospective, single center, cross-sectional	113 CD pts	children	PCR-SSO	HLA-DQ genotype	45/58/10
Viken et al. (2017) [46]	Norway	retrospective, multicenter, case-control	327 CD pts and 215 CD/T1DM pts	children	PCR-SSP	HLA-DQB1*0201 allele dose	141/321/78
Zubillaga et al. (2002) [47]	Spain	prospective, single center, cross-sectional	133 CD pts	children	PCR-SSP	HLA-DQB1*02 allele dose	63/63/7

CD: celiac disease; PCR-SSP: polymerase chain reaction with sequence-specific primers; PCR-SSO: polymerase chain reaction with sequence-specific oligonucleotide probes, Pts: patients RT-PCR: real-time polymerase chain reaction; T1DM: type 1 diabetes mellitus.

**Table 2:** Results of meta-analysis.

Outcomes, subgroups by age	Double vs. single dose of HLA-DQB1*02			Double vs. zero dose of HLA-DQB1*02		
	N^0^ of patients	OR (95% CI), *p*-value	Heterogeneity (I^2^, chi^2^)	N^0^ of patients	OR (95% CI), *p*-value	Heterogeneity (I^2^, chi^2^)
Atrophic vs. non-atrophic	722	0.991 (0.406–2.420), *p* = 0.984	11.8%, *p* = 0.338	430	2.626 (1.060–6.505), *p* = 0.037*	21.3%, *p* = 0.260
children	237	1.729 (0.319–9.370), *p* = 0.525	71.6%, *p* = 0.061*	159	1.757 (0.236–13096), *p* = 0.583	0.0%, *p* = 0.542
adults	379	0.537 (0.175–1.652), *p* = 0.278	0.0%, *p* = 0.682	200	2.534 (0.675–9.507), *p* = 0.168	0.0%, *p* = 0.945
Marsh 3c vs. Marsh 3a-b	862	0.870 (0.514–1.470), *p* = 0.602	39.7%, *p* = 0.127	418	0.822 (0.333–2.032), *p* = 0.671	46.8%, *p* = 0.068*
children	399	0.821 (0.401–1.681), *p* = 0.590	0.0%, *p* = 0.397	251	0.975 (0.296–3.208), *p* = 0.967	65.2%, *p* = 0.035*
adults	442	0.957 (0.420–2.184), *p* = 0.918	82.4%, *p* = 0.017*	147	0.753 (0.157–3.599), *p* = 0.722	50.2%, *p* = 0.134
Classical vs. non-classical	458	1.758 (1.148–2.692), *p* = 0.009*	0.0%, *p* = 0.744	221	1.701 (0.725–3.991), *p* = 0.222	40.7%, *p* = 0.168
children	305	2.082 (1.189–3.646), *p* = 0.010*	0.0%, *p* = 0.609	81	3.139 (1.142–8.630), *p* = 0.027*	0.0%, *p* = 0.747
Diarrhea vs. non-diarrhea	934	1.147 (0.863–1.523), *p* = 0.345	0.0%, *p* = 0.860	421	1.092 (0.655–1.818), *p* = 0.337	0.0%, *p* = 0.856
children	616	1.143 (0.819–1.593), *p* = 0.432	0.0%, *p* = 0.727	308	1.111 (0.569–2.170), *p* = 0.758	0.0%, *p* = 0.724
adults	318	1.158 (0.671–1.998), p = 0.599	0.0%, *p* = 1.000	113	1.065 (0.484–2.342), *p* = .0875	0.0%, *p* = 1.000
Type 1 diabetes mellitus	840	0.914 (0.437–1.912), *p* = 0.811	71.8%, *p* = 0.006*	411	1.169 (0.410–3.331), *p* = 0.770	89.8%, *p*<0.001*
children	766	0.597 (0.218–1.634), *p* = 0.315	79.3%, *p* = 0.008*	365	0.242 (0.045–1.312), *p* = 0.100	79.4%, *p* = 0.008*
		MD (95% CI), *p*-value	Heterogeneity (I^2^, chi^2^)		MD (95% CI), *p*-value	Heterogeneity (I^2^, chi^2^)
Age at diagnosis	512	-0.523 (-1.630 to 0.585), *p* = 0.355	28.6%, *p* = 0.231	147	-7.332 (-19.833 to 5.169), *p* = 0.250	71.4%, *p* = 0.015*
children	377	-0.303 (-1.156 to 0.551), *p* = 0.487	5.5%, *p* = 0.366	106	-2.026 (-5.824 to 1.771), *p* = 0.296	48.7%, *p* = 0.142
adults	133	-5.000 (-10.876 to 0.876), *p* = 0.095	0.0%, *p* = 1.000	41	-15.000 (-25.509 to -4.491), *p* = 0.005*	0.0%, *p* = 1.000

Asterisks indicate a *p*<0.05 for OR and MD, and a *p*<0.10 for heterogeneity tested with chi^2^-test. CI: confidence interval; OR: odds ratio; MD: mean difference.

### Clinical presentation

**Fig 2:**
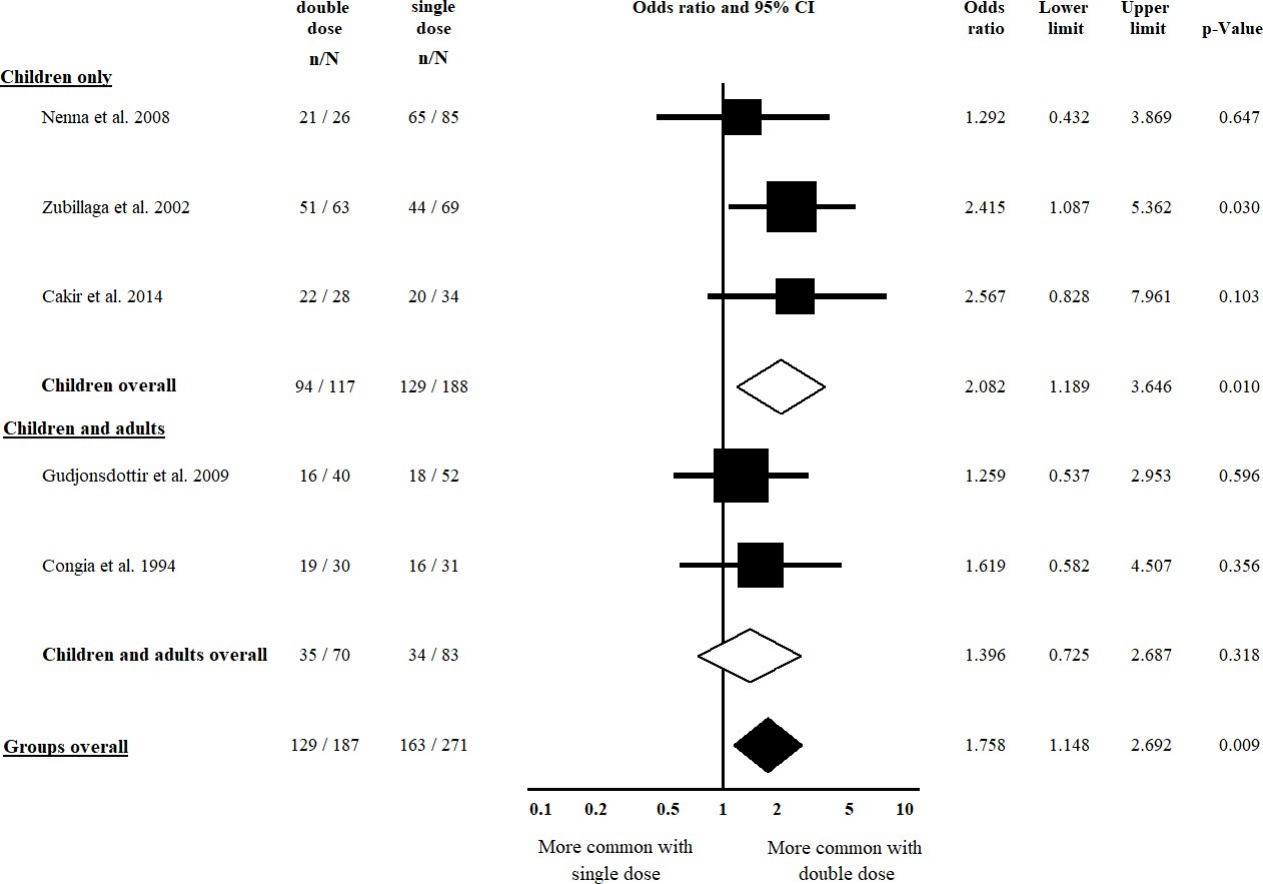
Odds ratios of classical presentation of CD at diagnosis with double dose vs. single dose of HLA-DQB1*02. Patients with a double dose of HLA-DQB1*02 had classical CD more frequently compared to those having a single dose. This association was more prominent in children. Heterogeneity of the groups overall: I^2^ = 0.0%, *p* = 0.744; heterogeneity of the subgroup of children: I^2^ = 0.0%, *p* = 0.609. CI: confidence interval.

**Fig 3:**
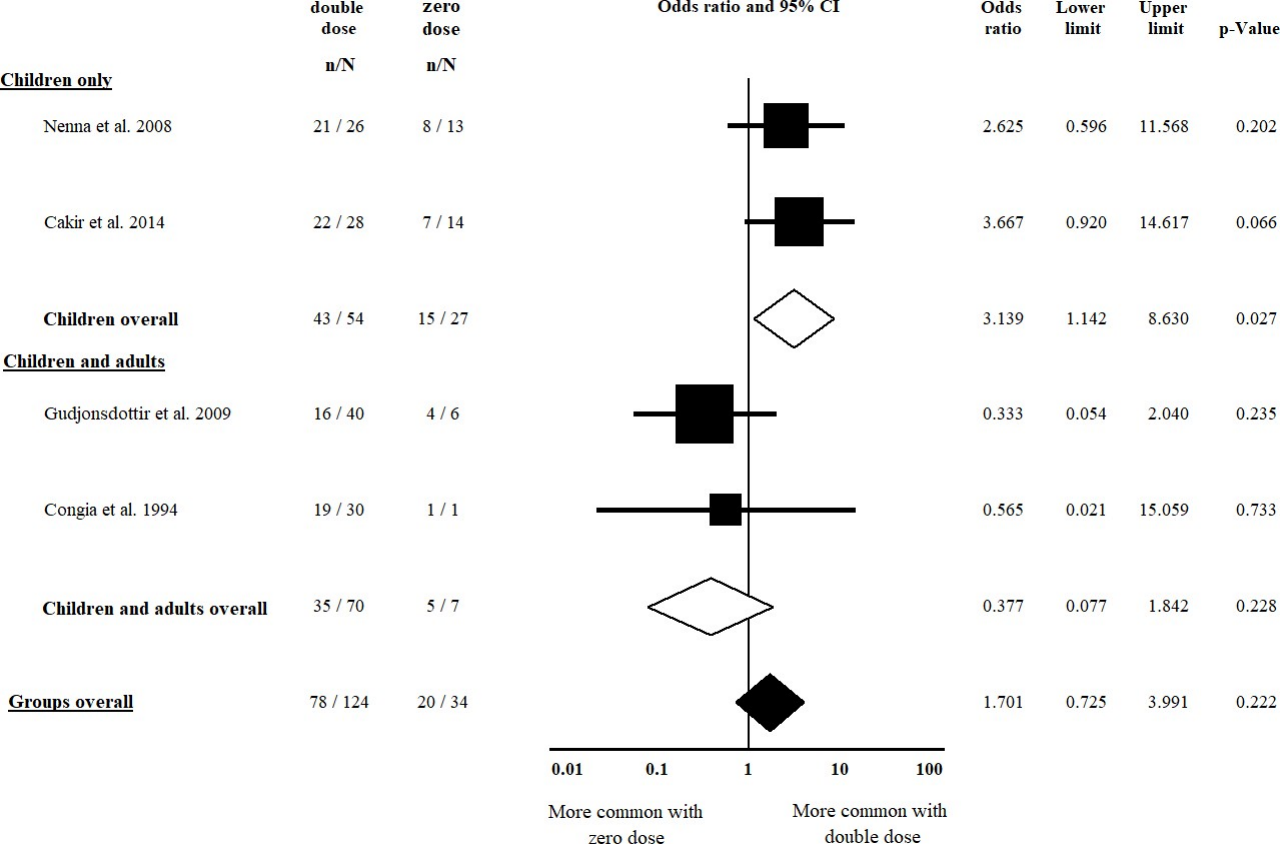
Odds ratios of classical presentation of CD at diagnosis with double dose vs. zero dose of HLA-DQB1*02. A significant gene dose effect was detected in the subgroup of children. Heterogeneity of the groups overall: I^2^ = 40.7%, *p* = 0.168; heterogeneity of the subgroup of children: I^2^ = 0.0%, *p* = 0.747. CI: confidence interval.

Five studies [22, 23, 38, 43, 47] were included in the comparison of classical vs. non-classical CD regarding double vs. single dose of HLA-DQB1*02; of them, four [22, 23, 38, 43] were included in the analysis regarding double vs. zero dose of HLA-DQB1*02. Patients with a double dose of HLA-DQB1*02 had classical CD more frequently, compared to those having a single dose of the allele (OR = 1.758, CI: 1.148 to 2.692, *p* = 0.009) in a homogeneous dataset (I^2^ = 0.0%, *p* = 0.744) (Fig 2). The difference was more prominent in the subgroup of children (OR = 2.082, CI: 1.189 to 3.646, *p* = 0.010) in a homogeneous dataset (I^2^ = 0.0%, *p* = 0.609) (Fig 2). In the analysis of a double dose of HLA-DQB1*02 vs. a zero dose of the allele, we detected a significant gene dose effect only if children were included in the analysis (OR = 3.139, CI: 1.142 to 8.630, *p* = 0.027) in a homogeneous dataset (I^2^ = 0.0%, *p* = 0.747) (Fig 3). Setting up the subgroup of children reduced the heterogeneity from 40.7% to 0.0% (Fig 3).

Five studies [24, 26, 28, 40, 44] reported on the presence of diarrhea at diagnosis, all were included in the analysis of double vs. single dose of HLA-DQB1*02 and that of double vs. zero dose of the allele, as well. We failed to detect a significant gene dose effect, nor in the subgroups of children and adults in a homogeneous dataset (I^2^ = 0.0%) (Table 2, S1 and S2 Figs).

### Age at diagnosis

Four [28, 36, 41, 43] and five studies [28, 36, 41, 43, 47] were included in the comparison of double vs. zero dose of HLA-DQB1*02 and double vs. single dose of the allele concerning age at diagnosis, respectively. Patients with a double dose of HLA-DQB1*02 were similar in age at diagnosis, compared to their counterparts with single and zero doses of the allele (MD: -0.523, CI: -1.630 to 0.585, *p* = 0.355; I^2^ = 28.60% and MD: -7.332, CI: -19.833 to 5.169, *p* = 0.250; I^2^ = 71.4% respectively) (S3 and S4 Figs). In the subgroup of children, heterogeneity was considerably reduced (Table 2).

### Histology at diagnosis

**Fig 4:**
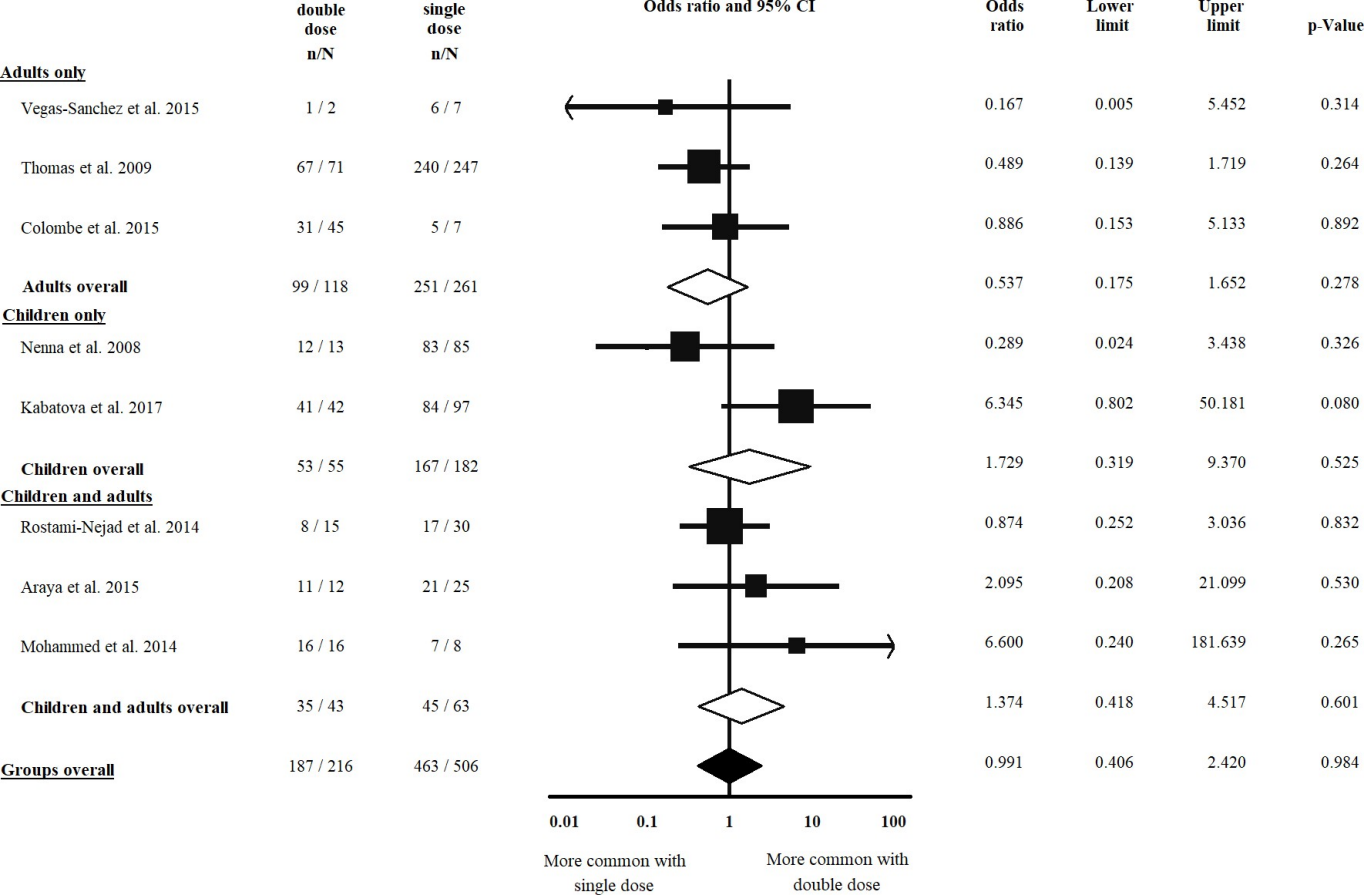
Odds ratios of atrophic histology at diagnosis with double dose vs. single dose of HLA-DQB1*02. We failed to detect a significant gene dose effect regarding diagnostic histology. Heterogeneity of the groups overall: I^2^ = 11.8%, *p* = 0.338; heterogeneity of the subgroup of adults: I^2^ = 0.0%, *p* = 0.682; heterogeneity of the subgroup of children: I^2^ = 71.6%, p = 0.061. CI: confidence interval.

**Fig 5:**
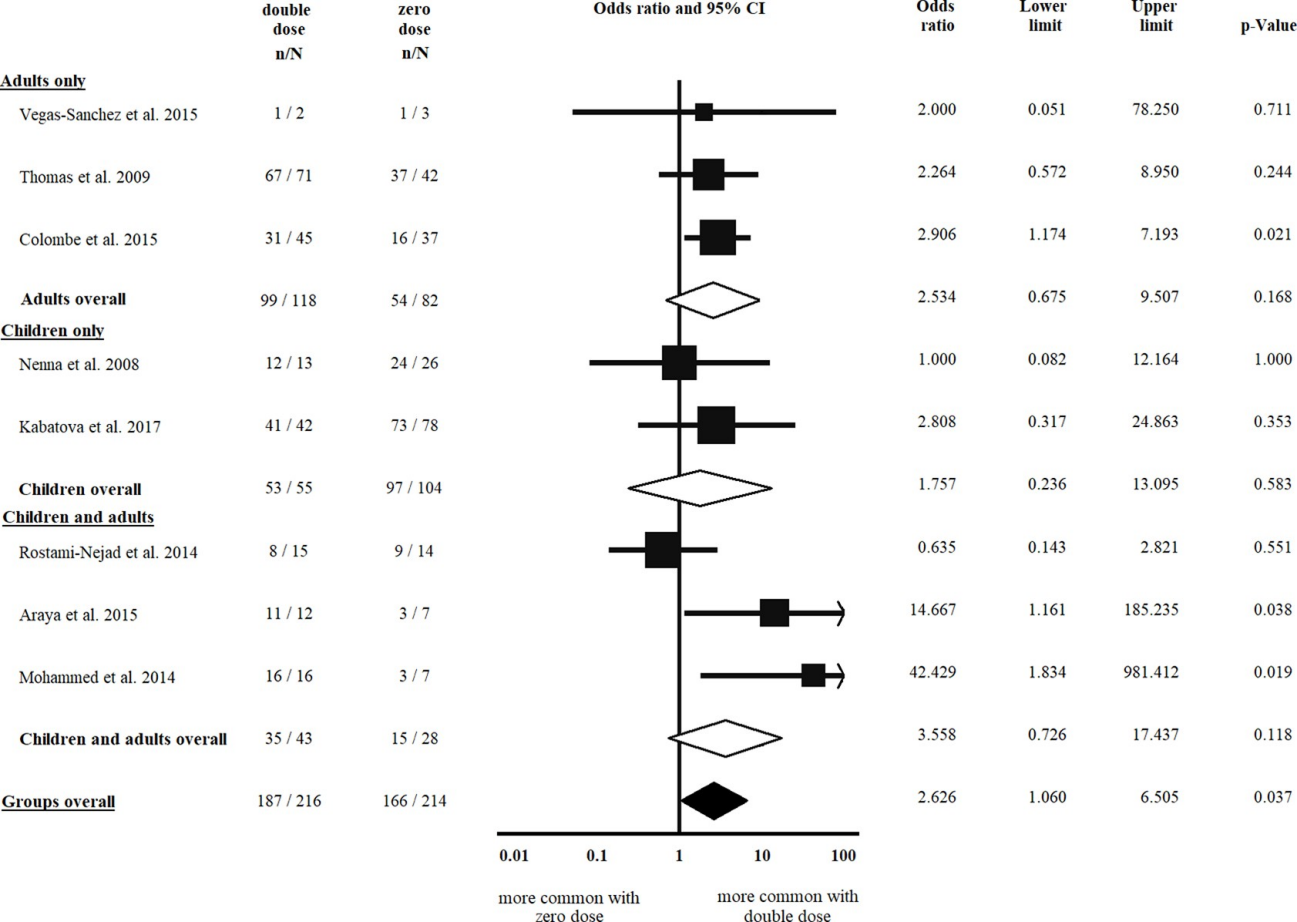
Odds ratios of atrophic histology at diagnosis with double dose vs. zero dose of HLA-DQB1*02. Patients with a double dose of the allele were more likely to have villous atrophy at diagnosis than those with a single dose of the allele. Heterogeneity of the groups overall: I^2^ = 21.3%, *p* = 0.260; heterogeneity of the subgroup of adults: I^2^ = 0.0%, *p* = 0.945; heterogeneity of the subgroup of children: I^2^ = 0.0%, *p* = 0.542. CI: confidence interval.

Eight studies [8, 19, 25, 37, 42–45] were eligible for inclusion in the comparison of atrophic vs. non-atrophic histology, all were included in the analysis of double vs. single dose of HLA-DQB1*02 and that of double vs. zero dose of the allele, as well. Villous atrophy at diagnosis was not more frequent in patients with a double dose of HLA-DQB1*02, as compared to a single dose of the allele (OR = 0.991, CI: 0.406 to 2.420, *p* = 0.984) in a homogeneous dataset (I^2^ = 11.8%, *p* = 0.338) (Fig 4). In contrast, patients with a double dose of the allele were more likely to have villous atrophy at diagnosis than those with a zero dose of the allele (OR = 2.626, CI: 1.060 to 6.505, *p* = 0.037) (Fig 5). The subgroup analysis of children and adults did not result in significances across groups (Figs 4 and 5, Table 2).

Regarding the severity of villous atrophy, seven [12, 24, 25, 41–44] and eight studies [12, 24, 25, 41–45] were eligible for inclusion in the analysis of double vs. single dose of HLA-DQB1*02 and that of double vs. zero dose of the allele. Marsh 3c at diagnosis was not more frequent in patients with a double dose of HLA-DQB1*02, as compared to single and zero doses of the allele (OR = 0.870, CI: 0.514 to 1.470, *p* = 0.602 [I^2^ = 39.7%, *p* = 0.127] and OR = 0.822, CI: 0.333 to 2.032, *p* = 0.671 [I^2^ = 46.8%, *p* = 0.068], respectively). These remained unchanged in the subgroups of children and adults (S5 and S6 Figs, Table 2).

### Type 1 diabetes mellitus

Five [20, 21, 27, 39, 46] studies were included in the analysis of double vs. single dose of HLA-DQB1*02; of them, four [20, 21, 27, 46] were eligible for inclusion in the analysis of double vs. zero dose of the allele. Analyses revealed no significant gene dose effect concerning the coexistence of type 1 diabetes mellitus and CD, which remained unchanged in the subgroups of children and adults. All analyses suffered from significant heterogeneity (S7 and S8 Figs, Table 2).

### Other patient and disease characteristics

There were several reports on the association between HLA-DQ2 gene dose effect and the clinical phenotype of CD (age at onset, anemia, serology, autoimmunity, body mass index, osteoporosis, oral manifestations, complicated CD, and dermatitis herpetiformis). Data reported were insufficient for quantitative synthesis. We report the studies and the direction of associations with HLA-DQ gene dose in Table 3.

**Table 3:** Summary of studies reporting on gene dose effect.

Characteristics	Association between HLA-DQ2 gene dose and the clinical phenotype		
	Positive	No association	Negative
Clinical presentation	[38, 41, 43, 47, 51, 66, 69, 79]	[7, 12, 23, 26, 28, 36, 40, 44, 61, 71, 72, 74]	
Age at onset	[47]	[26, 40, 45, 53, 71, 73, 75–77]	
Age at diagnosis	[38, 41, 47, 66]	[28, 36, 40, 44, 71, 72]	[13, 68]
Histology at diagnosis	[12, 37, 41, 43, 78]	[7, 28, 36, 44, 53, 69, 71]	
Anemia	[41]	[26, 36, 40, 43, 44]	
Serology	[7, 42, 43, 67, 69, 70]	[36, 44, 48, 59, 65, 71, 78]	
Autoimmunity	[27, 39, 62]	[64]	[46, 54]
Body mass index		[26, 44]	
Osteoporosis		[44]	
Oral manifestations (DED, RAS)	[55, 63]	[44]	
Complicated disease (RCD, EATL, SBC)	[14, 49, 50, 64]	[58]	
Dermatitis herpetiformis			[57]

DED: dental enamel defect; EATL: enteropathy-associated T-cell lymphoma; RAS: recurrent aphthous stomatitis; RCD: refractory celiac disease; SBC: small bowel carcinoma.

### Publication bias

Although funnel plots seem symmetric, the low number of studies raised concerns about an uncertain assessment of symmetry (S3 Appendix).

### Sensitivity analysis

When we omitted studies one-by-one, there was no change in the direction of the main association, except for two outcomes. Omitting Zubillaga et al. from the analysis on classic vs. non-classic clinical presentation resulted in the loss of statistical significance in the comparison of double vs. single dose. Omitting Vermeulen et al. from the analysis on age at diagnosis, resulted in a significant gene dose effect (MD = -0.248, CI: -0.464 to -0.032, *p* = 0.024) in the comparison of double vs. single dose.

### Risk of bias assessment

**Fig 6:**
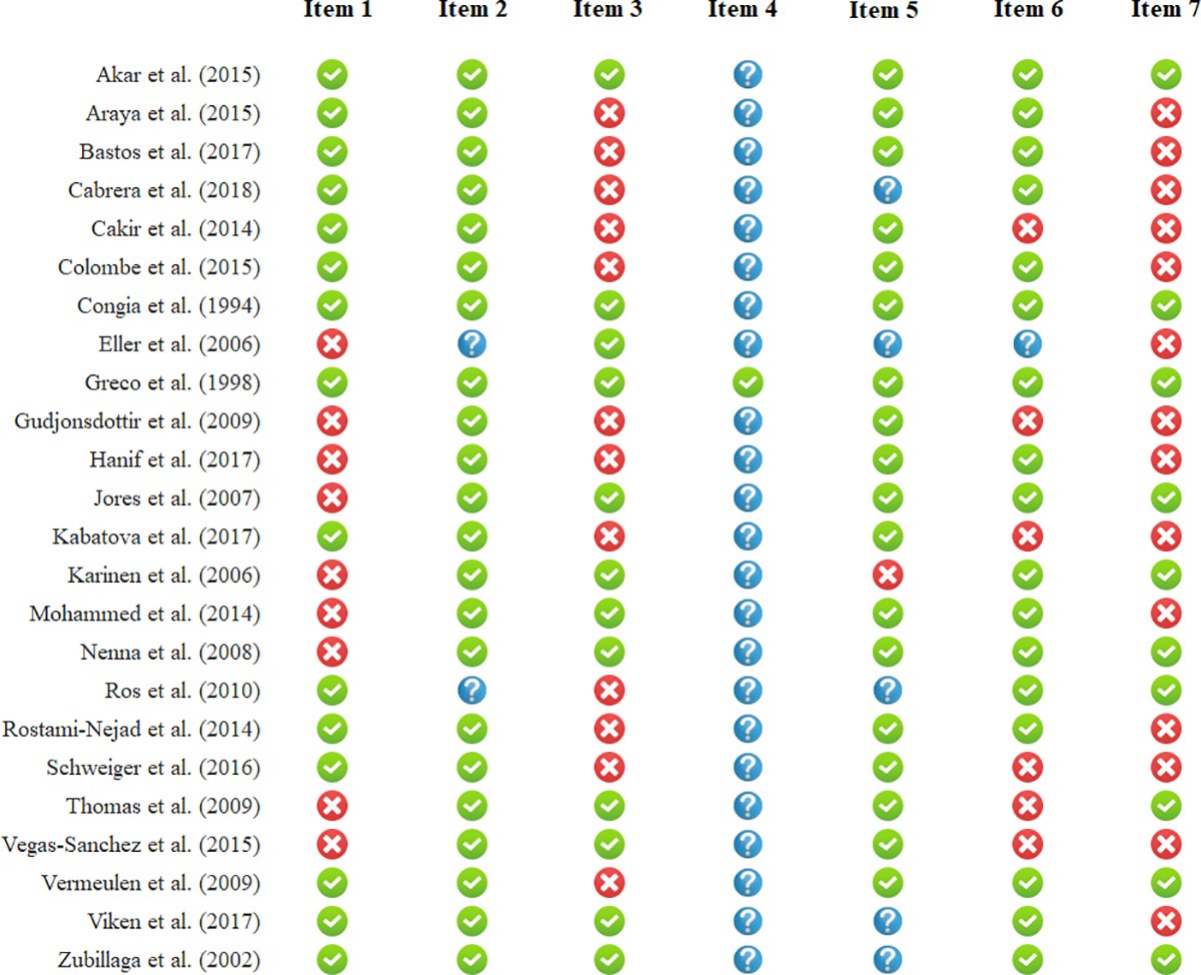
Summary of risk of bias in individual studies included in meta-analysis. Green, red, and blue icons represent low, high, and uncertain risk of bias. Definitions of items are provided in S1 Table.

Results of risk of bias assessment are presented in Fig 6. Ten out of 24 studies (41.7%) aimed to analyze gene dose effect primarily. Twenty-two out of 24 (91.7%) and 18 out of 24 (75.0%) studies used a low-risk classification for diagnosing CD and rating diagnostic histology, respectively. However, the average CD population was clearly represented only in 62.5% of the studies included. Appropriate blinding was applied in only one study (4.2%).

## Discussion

We aimed to review the current knowledge about the influence of HLA-DQB1*02 gene dose on the phenotype of celiac disease in our study.

Although reasonable molecular mechanisms have been proposed by *in vitro* experiments [5, 6], gene dose effect seemed to influence only the clinical phenotype defined by Oslo criteria and histology. Patients with a double dose of HLA-DQB1*02 exhibited more often classical phenotype and villous atrophy, as compared to those with a single dose of the allele, whereas no evidence of gene dose effect was collected on diarrhea, age at diagnosis, the severity of villous atrophy, and the frequency of type 1 diabetes mellitus (Table 2).

Lack of clinically significant gene dose effect is also supported by experimental results, as well: (1) an equal magnitude of specific T-cell responses characterizes homo- and heterozygotes, (2) the amount of DQA1*05 and DQB1*02 mRNS in heterozygotes exceeded the expected 50% of that measured in homozygotes [80]. Other factors should be taken into consideration, e.g., complementing HLA risk stratification with ten non-HLA loci changed the allocation of 10% of study population from the moderate- to the high-risk group [81]. The effect of non-HLA loci may outweigh that of HLA. In addition, non-genetic (environmental) factors appear to contribute to CD phenotype [23, 28, 40].

Concerning comorbid conditions, data allowed us to perform analysis only on type 1 diabetes mellitus, which is the most frequent co-existing immune-mediated disorder in CD. We failed to observe a significant gene dose effect in the frequency of type 1 diabetes mellitus. This corroborates with previous studies confirming that the combined occurence of DQ2 and DQ8 is high in patients with both CD and type 1 diabetes mellitus [27, 46, 54].

Although we were unable to meta-analyze other disease complications due to discrepancies in reporting, the importance of HLA-DQ2 gene dose might be highlighted by these studies (Table 3). HLA status appears to be important in the development of malignant complications. Lymphomatous complications were more frequent in patients with a double dose of HLA-DQ2 in a study [64]. In a prospective study conducted by Al-Toma et al., HLA-DQ2.5 homozygosity was associated with serious CD complications, namely, type 2 refractory celiac disease and enteropathy-associated T-cell lymphoma [14]. Prevalence of DQ2 homozygosity was significantly higher in patients with a complicated disease (i.e., patients with type 1 and type 2 refractory CD, EATL, or SBC), as compared to those with non-complicated CD in a retrospective Italian and a multicentre study [49, 50]. However, it is noteworthy that data of Howell et al. did not confirm these observations [58]. Further studies aiming to resolve these controversies are awaited to test whether determining HLA gene dose is appropriate for risk stratification for severe complications.

### Strengths and limitations

To date, no meta-analyses have investigated HLA-DQB1*02 gene dose effect in CD. Our main strength is the transparent and comprehensive search and the rigorous selection process; however, we must acknowledge that the evidence is limited due to a number of reasons.

We did not contact the original authors of the included or excluded papers to acquire further information; only published material was used to preserve reproducibility.

Data on the clinical phenotype of celiac disease were collected from patients’ files retrospectively. Regarding HLA status, HLA-typing was performed (1) at the time of enrolment to the study (at or after the diagnosis of celiac disease) or (2) before enrolment (retrospectively collected from charts). In relation to the outcomes, both ways are retrospective. However, HLA is a genetic marker, an unchangeable feature of the patients. Therefore, in our opinion, timing of HLA-typing does not affect data quality.

Design of the studies included were (1) controlled studies including celiac and non-celiac subjects (case-control or cross-sectional studies) or (2) uncontrolled case series reports of celiac patients. In this meta-analysis, data on celiac patients were desirable, exclusively (while the non-celiac groups were irrelevant for us). As a consequence, there were studies included not aiming to analyze gene dose effect, as a primary objective, which may limit the data reported from the studies.

The number of eligible studies was low, not allowing us to draw reliable conclusions from the symmetry of the funnel plots to assess small-study effect (S3 Appendix).

Relevant clinical questions as to whether gene dose effects anemia, complications of CD, or serology have not been meta-analyzed due to incoherent data reporting across studies (S2 Table).

As only nine studies typed HLA-DQB1*0201 [12, 36–39, 41, 44–46] (the rest of them typed DQB1*02 allele [40, 42, 43, 47]), our conclusion is not generalizable to the role of HLA-DQB1*0201 which may be of greater clinical importance. DQB1*02 allele dose was calculated from DQ2 haplotype-based HLA risk stratification systems in eleven studies [8, 19–28].

Self-reported clinical symptoms (e.g., bloating, diarrhea, and abdominal discomfort) are difficult to quantify objectively. To minimize the distortion, we used the widely accepted Oslo classification (i.e., dichotomization of patients into classical [with malabsorption] and non-classical [without malabsorption] phenotypes) [16].

Some analyses suffer from statistical heterogeneity (Table 2) which can be explained by methodological heterogeneity (i.e., histological assessment, HLA-typing, inclusion and exclusion of subjects) and clinical heterogeneity (i.e., geography, diverse environmental factors [e.g., timing of gluten introduction], and genetics [i.e., HLA and non-HLA loci]). A part of heterogeneity may originate from the age of participants: heterogeneity reduced when analyzing data from children and adults separately in some analysis, while persisted in others (Table 2).

## Conclusion

### Implications for clinical practice

Our results suggest a significant gene dose effect regarding clinical presentation: classical clinical presentation and villous atrophy are more frequent in patients with a double dose of HLA-DQB1. We were unable to prove a similar effect in terms of diarrhea at diagnosis, age at diagnosis, the degree of atrophy, and type 1 diabetes mellitus. Recent guidelines do not require HLA-typing to set up the diagnosis of CD apart from pediatric cases diagnosed without intestinal biopsy. The role of it is mainly restricted to the exclusion of CD [82, 83]. Patients with high-risk HLA status may be at higher risk of severe disease course, raising concerns about the need for a stricter gluten-free diet and follow-up. However, these results should be treated with caution due to the limitations of the data available for our study.

### Implications for research

Studies validating our results and investigating the association between gene dose effect and disease complications (e.g., malignant tumors [EATL, SBC], autoimmune disorders, and RCD) are awaited.

## Supporting information

S1 Table Risk of bias assessment with the modified Newcastle-Ottawa scale. (DOCX)

S2 Table Papers eligible for quantitative synthesis but not included in meta-analysis. (DOCX)

S3 Table Data included in meta-analysis. (XLSX)

S1 Fig Odds ratios of diarrhea at diagnosis of celiac disease with double dose vs. single dose of HLA-DQB1*02. CI: confidence interval.
(DOCX)

S2 Fig Odds ratios of diarrhea at diagnosis of celiac disease with double dose vs. zero dose of HLA-DQB1*02. CI: confidence interval.
(DOCX)

S3 Fig Mean difference of age at diagnosis of celiac disease with double dose vs. single dose of HLA-DQB1*02. CI: confidence interval.
(DOCX)

S4 Fig Mean difference of age at diagnosis of celiac disease with double dose vs. zero dose of HLA-DQB1*02. CI: confidence interval.
(DOCX)

S5 Fig Odds ratios of total villous atrophy at diagnosis of celiac disease with double dose vs. single dose of HLA-DQB1*02. CI: confidence interval.
(DOCX)

S6 Fig Odds ratios of total villous atrophy at diagnosis of celiac disease with double dose vs. zero dose of HLA-DQB1*02. CI: confidence interval.
(DOCX)

S7 Fig Odds ratios of type 1 diabetes with double dose vs. single dose of HLA-DQB1*02. CI: confidence interval.
(DOCX)

S8 Fig Odds ratios of type 1 diabetes with double dose vs. zero dose of HLA-DQB1*02. CI: confidence interval.
(DOCX)

S1 Appendix PRISMA checklist. (DOC)

S2 Appendix Draft of search. (TXT)

S3 Appendix Publication bias (funnel plots). (DOCX)
