# Supplementary material for: Classical celiac disease is more frequent with a double dose of HLA-DQB1*02: A systematic review with meta-analysis
Source: PLoS One. 2019 Feb 14;14(2):e0212329. doi: 10.1371/journal.pone.0212329 (PMC6375622; doi:10.1371/journal.pone.0212329)
Supplement: S1 Table — (DOCX) [file pone.0212329.s001.docx]

**S1 Table. Risk of bias assessment - definitions of items. The modified Newcastle-Ottawa Scale (NOS)**

The Newcastle-Ottawa Scale (NOS) tool, dedicated to assessing cohort studies, was adjusted to the design of the studies answering to the review question. Quality was assessed by two investigators unblinded to publication data who judged each study by pre-defined items. The modified scale consists of 7 items. Items were rated as ‘high risk’, ‘low risk’, or ‘unclear risk’ corresponding to the definitions (as detailed below). We decided not to compile quality scores arbitrarily.

**Items of quality assessment**

**Item 1, representativeness:** the item concerns the generalizability of the findings to the average celiac population

✅*Low risk:* non-selected celiac patients (consecutive or random selection). Reasonable exclusions carrying a low risk of bias: patients with an uncertain diagnosis of celiac disease (i.e., differential diagnostic exclusions, such as giardiasis, olmesartan-induced enteropathy).

✖*High risk:* unjustified exclusions or exclusions indicating high risk of bias

**?***Unknown risk:* data on the item is not reported

**Item 2, diagnosis of celiac disease**

✅*Low risk:* patients were diagnosed by the current guidelines (biopsy-proven disease)

✖*High risk:* patients were not diagnosed by the current guidelines

**?***Unknown risk:* no data reported

**Item 3,** **HLA-risk stratification**

✅*Low risk:* HLA-DQB1*02 gene dose was determined and published within the study

✖*High risk:* HLA-DQB1*02 gene dose was determined by the review authors based on HLA-risk groups published within the study

**?***Unknown risk:* data on the item is not reported

**Item 4,** **blinding:** the item concerns the blinding of the person(s) to gene dose, who rate(s) the clinical symptoms

✅*Low risk:* appropriate blinding

✖*High risk:* unblinded design

**?***Unknown risk:* data on the item is not reported

**Item 5,** **definitions of outcomes: diagnostic histology**

✅*Low risk:* the study used Marsh, Marsh-Oberhuber, or Corazza classification to rate intestinal histology. Vh:Crd<2(3):1 was defined as the cut-off value between atrophic and non-atrophic histology

✖*High risk:* the study used any other classification as mentioned above or the chosen Vh:Crd rate is likely to introduce bias

**?***Unknown risk:* data on the item is not reported

**Item 6, attrition**: the item indicates the difference between the number of patients included in the study and that of patients included in the analysis or whom data were reported on

✅*Low risk:* no or minimal attrition unlikely to introduce bias

✖*High risk:* considerable attrition likely to introduce bias

**?***Unknown risk*: no data about the loss

**Item 7, primary objective of the study**:

✅*Low risk:* the primary objective of the study was to determine the association between HLA-DQ2 gene dose and clinical outcomes

✖*High risk:* the primary objective does not correspond to the ‘low risk’ definition

**?***Unknown risk*: no data about primary objective
