# Supplementary material for: Classical celiac disease is more frequent with a double dose of HLA-DQB1*02: A systematic review with meta-analysis
Source: PLoS One. 2019 Feb 14;14(2):e0212329. doi: 10.1371/journal.pone.0212329 (PMC6375622; doi:10.1371/journal.pone.0212329)
Supplement: S2 Table — (DOCX) [file pone.0212329.s002.docx]

**S2 Table. Papers eligible for quantitative synthesis but not included in meta-analysis**

| **Author (year)** | **Country** | **Settings** | **N^O^ of pts.** | **Age group** | **HLA-typing** | | | **Outcomes reported** | **Reasons for exclusion** |
| --- | --- | --- | --- | --- | --- | --- | --- | --- | --- |
|  |  |  |  |  | **Method** | **Target of typing within the study** | **N^0 of^ pts. (double/single/zero)** |  |  |
| Agardh et al. (2015) | multinational | prospective, multicenter, observational | 340 CD pts | children | PCR | HLA-DQ2 genotype | not reported | clinical presentation | Lack of comparability of data about gene dose |
| Al-Toma et al. (2006) | The Netherlands | retrospective, single center | 116 complicated CD pts and 121 uncomplicated CD pts | adults | PCR-SSP | HLA-DQ2 genotype | 71/145/20 | complications (RCD1, RCD2, EATL) | Lack of comparability of outcomes |
| Biagi et al. (2014) | Italy | retrospective, multicenter, case-control | 116 complicated CD pts and 181 uncomplicated CD pts | adults | PCR-SSP, SSO | HLA-DQ2 genotype | 65/140/0 | complications (RCD1, RCD2, EATL, small bowel carcinoma) | Lack of comparability of outcomes |
| Biagi et al. (2012) | Italy | retrospective, single center, case-control | 169 uncomplicated CD pts, 27 complicated CD pts, 22 potential CD pts, 224 healthy stem cell donor | adults | PCR-SSP, SSO | HLA-DQB1*02 allele dose | not reported | complications (RCD1, RCD2, EATL, small bowel carcinoma) | Lack of comparability of data about gene dose and missing numerical data |
| Celestino et al. (abstract) (2011) | Italy | retrospective, single center, cross-sectional | 122 CD pts | children and adults | not reported | HLA-DQB1*02 allele dose | not reported | clinical presentation | Lack of comparability of data about gene dose |
| Ceylan et al. (2014) | Turkey | retrospective, single center, cross-sectional | 40 CD pts | adults | PCR-SSP | HLA-DQB1*0201 allele dose | not reported | histology | Lack of comparability of data about gene dose |
| Constantinidou et al. (abstract) (2009) | Greece | retrospective, single center, case control | 67 CD pts and 120 control | children | PCR-SSO and SSP | HLA-DQB1*02 allele dose | 43/17/7 | age at onset, histology | Missing numerical data |
| Delgado et al. (2014) | Spain | retrospective, single center, cross-sectional | 91 CD pts | children | PCR-SSP | HLA-DQ2 genotype | not reported | histology, serology | Lack of comparability of data about gene dose |
| Dezsőfi et al. (2008) | Hungary | retrospective, single center, case-control | 40 T1DM pts and 100 CD pts and CD/T1DM pts | children | PCR | HLA-DQ2 genotype | not reported | complication (T1DM) | Lack of comparability of data about gene dose |
| Erriu et al. (2013) | Italy | prospective, single center, cross-sectional | 44 CD pts | children | PCR | HLA-DQB1*02 allele dose | 17/18/9 | dental complications (DED, RAS) | Lack of comparability of data about the outcomes |
| Fernandez-Cavada Pollo et al. (abstract) (2012) | Spain | prospective, single center, case-control | 332 CD pts and 155 control | children and adults | PCR SSP and SSO | HLA-DQB1*02 allele dose | not reported | age (children vs. adult) | Missing numerical data |
| Ghawil et al. (2011) | Libya | prospective, single center, case control | 24 CD/T1DM and 194 T1DM pts | children | PCR-SSP | HLA-DQB1*0201 allele dose | 9/11/4 | age at onset | Lack of comparability of data about the outcomes |
| Hall et al. (1996) | UK | prospective, single center, case-control | 88 CD pts (23 with, 65 without DH) and healthy control | adults | PCR-SSO | HLA-DQB1*02 allele dose | 30/51/ 0 | complications (DH) | Lack of comparability of data about the outcomes |
| Howell et al. (1995) | UK | retrospective, multicenter, case-control | 91 CD pts and 43 EATL pts | children and adults | PCR-SSO | HLA-DQB1*0201 allele dose | 44/84/6 | complications (EATL) | Lack of comparability of data about the outcomes |
| Klein et al. (2005) | The USA | retrospective, single center | 116 CD pts | children and adults | not reported | HLA-DQ2 genotype | 22/82/12 | serology | Lack of comparability of data about the outcomes |
| Klimov et al. (abstract) (2017) | Russia | retrospective, multicenter, cross-sectional | 112 CD pts | children | PCR | HLA-DQ2 genotype | not reported | age at diagnosis | Lack of comparability of data about gene dose |
| Laadhar et al. (2009) | Tunisia | prospective, single center, case-control | 40 CD pts diagnosed according ESPGHAN criteria, 38 screening diagnosed CD pts and 40 healthy control | children | PCR-SSP | HLA-DQ2 genotype | not reported | clinical presentation | Lack of comparability of data about gene dose |
| Liu et al. (2014) | The USA, Finland, Sweden, Germany (TEDDY group) | prospective, multicenter | 291 CD pts | children | PCR | HLA-DQ2 genotype | not reported | complication (autoimmunity) | Lack of comparability of data about the outcomes |
| Majoranna et al. (2010) | Italy | prospective, single center, case-control | 250 CD pts and 125 healthy | children | PCR | HLA-DQ2 genotype | not reported | complications (DED) | Lack of comparability of data about the outcomes |
| Malamut et al. (2013) | France | retrospective, single center, cross-sectional | 222 CD pts | adults | not reported | HLA-DQ2 genotype | not reported | complications (autoimmune disease, lymphoproliferative complication) | Lack of comparability of data about the outcomes |
| Maxim et al. (abstract) (2018) | Romania | retrospective, single center | 75 CD pts | adults | PCR | HLA-DQB1*02 allele dose | not reported | serology | Lack of comparability of data about the outcomes |
| Maxim et al. (abstract) (2018) | Romania | retrospective, single center | 75 CD pts | adults | PCR | HLA-DQ2 genotype | not reported | age at diagnosis, clinical presentation | Lack of comparability of data about gene dose |
| Mills et al. (abstract) (2015) | The USA | prospective, single center, cross-sectional | 3136 tTGA positive pts | adults | high resolution DNA-typing | HLA-DQB1*02 gene dose | not reported | serology | Missing numerical data |
| Morreale et al. (abstract) (2016) | Italy | retrospective, multicenter, cross-sectional | 132 CD pts | adults | PCR | HLA-DQ2 genotype | not reported | age at diagnosis, clinical presentation, serology, anemia, histology | Lack of comparability of data about gene dose |
| Mubarak et al. (abstract) (2012) | The Netherlands | retrospective, single center, cross-sectional | 95 CD pts | children | PCR-SSO | HLA-DQB1*02 allele dose | not reported | serology | Lack of comparability of data about the outcomes |
| Murray et al. (2007) | The USA | prospective, single center, case-control | 84 CD pts and 102 healthy blood donor | adults | PCR-SSP | HLA-DQB1*02 allele dose | 34/79/3 | age at onset, age at diagnosis, clinical presentation, histology | Missing numerical data |
| Mustalahti et al. (2002) | Finland | prospective, single center, cross-sectional | 28 CD pts | adults | PCR-SSP | HLA-DQ2 genotype | not reported | age at diagnosis, clinical presentation | Missing numerical data |
| Pena-Quintana et al. (2003) | Spain | prospective, single center, case-control | 118 CD pts and 236 control | children | PCR-SSO | HLA-DQB1*02 allele dose | 47/65/6 | age at onset | Missing numerical data |
| Piccini et al. (2012) | Italy | prospective, single center, case-control | 89 CD pts and 70 healthy control | children | PCR-SSP | HLA-DQB1*02 allele dose | not reported | clinical presentation | Lack of comparability of data about gene dose |
| Ploski et al. (1993) | Norway | prospective, single center, case-control | 94 CD pts and 47 healthy control | children | PCR-SSO | HLA-DQB1*0201 allele dose | 46/43/5 | age at diagnosis | Lack of comparability of data about the outcomes |
| Polvi et al. (1996) | Finland | prospective, single center, case-control | 31 CD pts and 25 healthy sibs and 32 control | children and adults | PCR-SSO | HLA-DQB1*02 allele dose | not reported | age at onset | Missing numerical data |
| Senapati et al. (2016) | India | retrospective, single center | 531 CD children and 871 CD adults | children and adults | immunochip genotyping | HLA-DQ2-genotype | 87/330/78 | age (children vs. adult) | Lack of comparability of data about the outcomes |
| Tuysuz et al. (2001) | Turkey | prospective, single center, case control | 55 CD pts and 50 control | children | PCR-SSP | HLA-DQB1*02 allele dose | not reported | age at onset | Missing numerical data |
| Wu et al. (abstract) (2014) | The USA | retrospective, single center, cross-sectional, | 89 CD pts | adults | PCR | HLA-DQ2 genotype | not reported | clinical presentation, histology, serology, anemia | Lack of comparability of data about the outcomes and missing numerical data |
| Zamani et al. (abstract) (2014) | Iran | prospective, single center, case-control | 120 CD pts and 100 healthy control | children and adults | PCR-SSP | HLA-DQB1*0201 allele dose | not reported | clinical presentation | Missing numerical data |

CD: celiac disease; DED: dental enamel defect; DH: dermatitis herpetiformis; EATL: enteropathy associated T-cell lymphoma; pts: patients; PCR-SSP: polymerase chain reaction with sequence-specific primers; PCR-SSO: polymerase chain reaction with sequence-specific oligonucleotide probes; RAS: recurrent aphthous stomatitis; RCD: refractory celiac disease; SBC: small bowel carcinoma; tTGA: tissue transglutaminase antibody.
