# Supplementary material for: Classical celiac disease is more frequent with a double dose of HLA-DQB1*02: A systematic review with meta-analysis
Source: PLoS One. 2019 Feb 14;14(2):e0212329. doi: 10.1371/journal.pone.0212329 (PMC6375622; doi:10.1371/journal.pone.0212329)
Supplement: S2 Fig — CI: confidence interval. (DOCX) [file pone.0212329.s005.docx]

**S2 Figure. Odds ratios of diarrhea at diagnosis of celiac disease with double dose vs. zero dose of HLA-DQB1*02
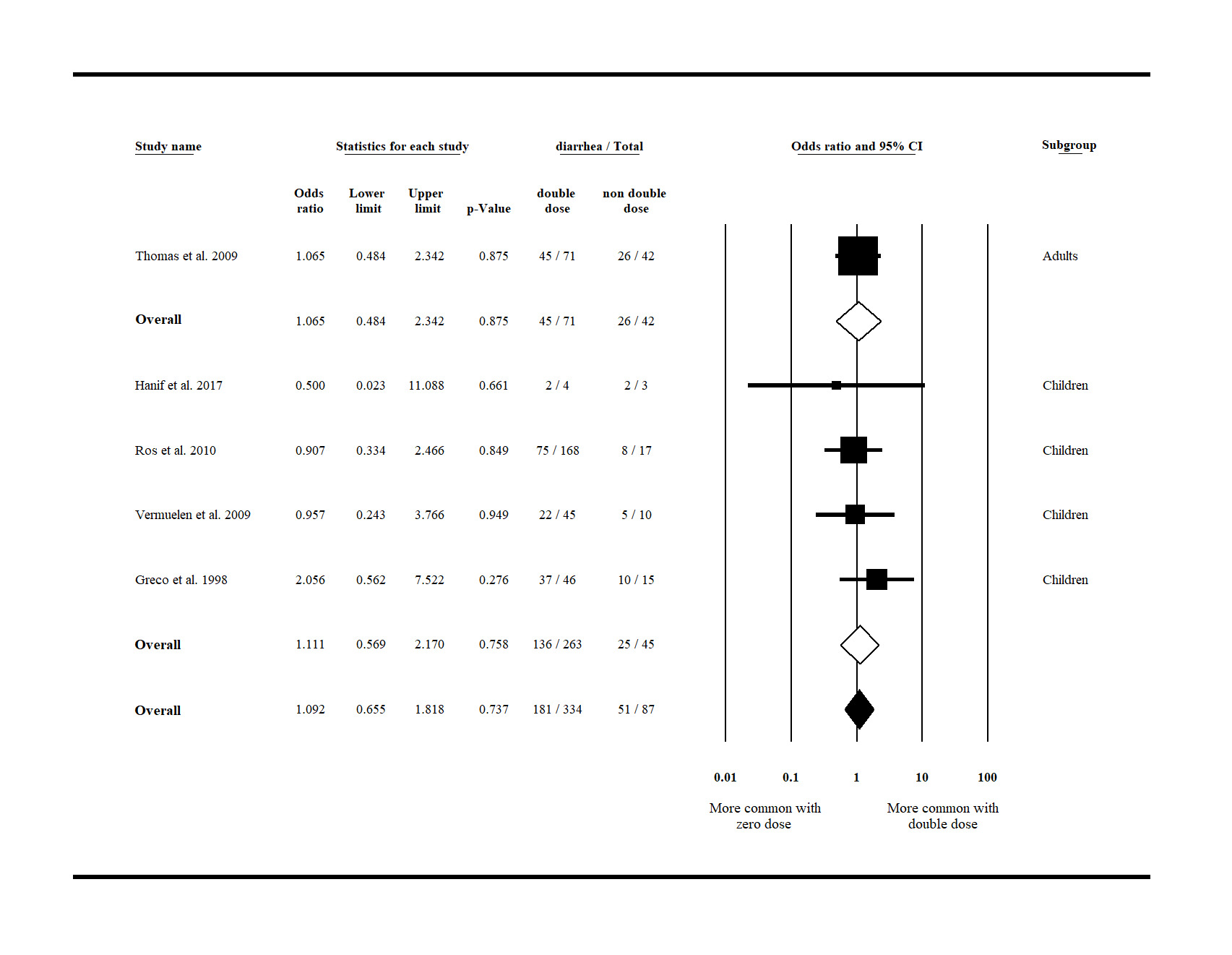
**
