# Supplementary figures and images for: Classical celiac disease is more frequent with a double dose of HLA-DQB1*02: A systematic review with meta-analysis
Source: PLoS One. 2019 Feb 14;14(2):e0212329. doi: 10.1371/journal.pone.0212329 (PMC6375622; doi:10.1371/journal.pone.0212329)

**S7 Figure.** **Odds ratios of type 1 diabetes with double dose vs. single dose of HLA-DQB1*02
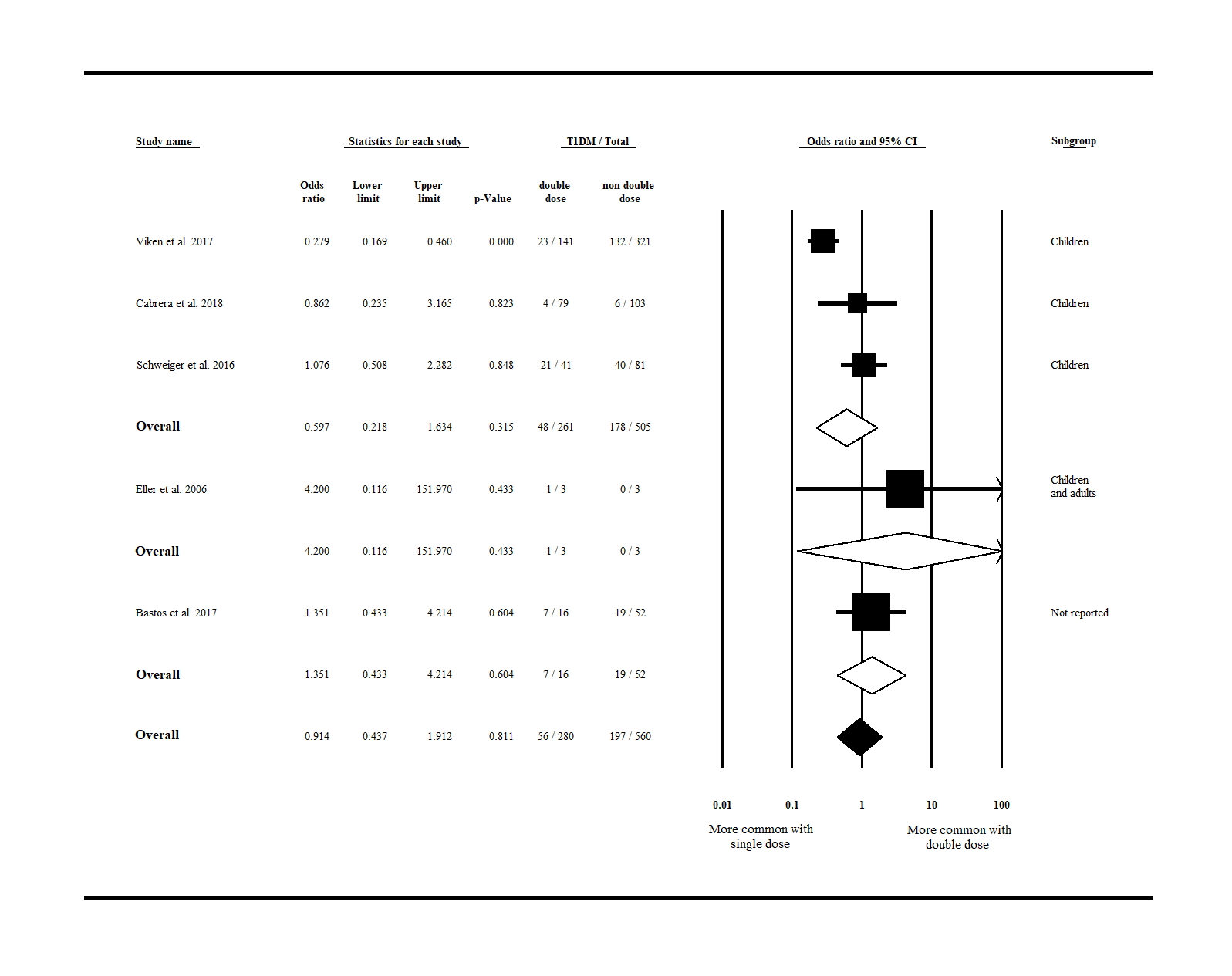
**

Supplement: S7 Fig — CI: confidence interval. (DOCX) [file pone.0212329.s010.docx]

**S8 Figure.** **Odds ratios of type 1 diabetes with double dose vs. zero dose of HLA-DQB1*02
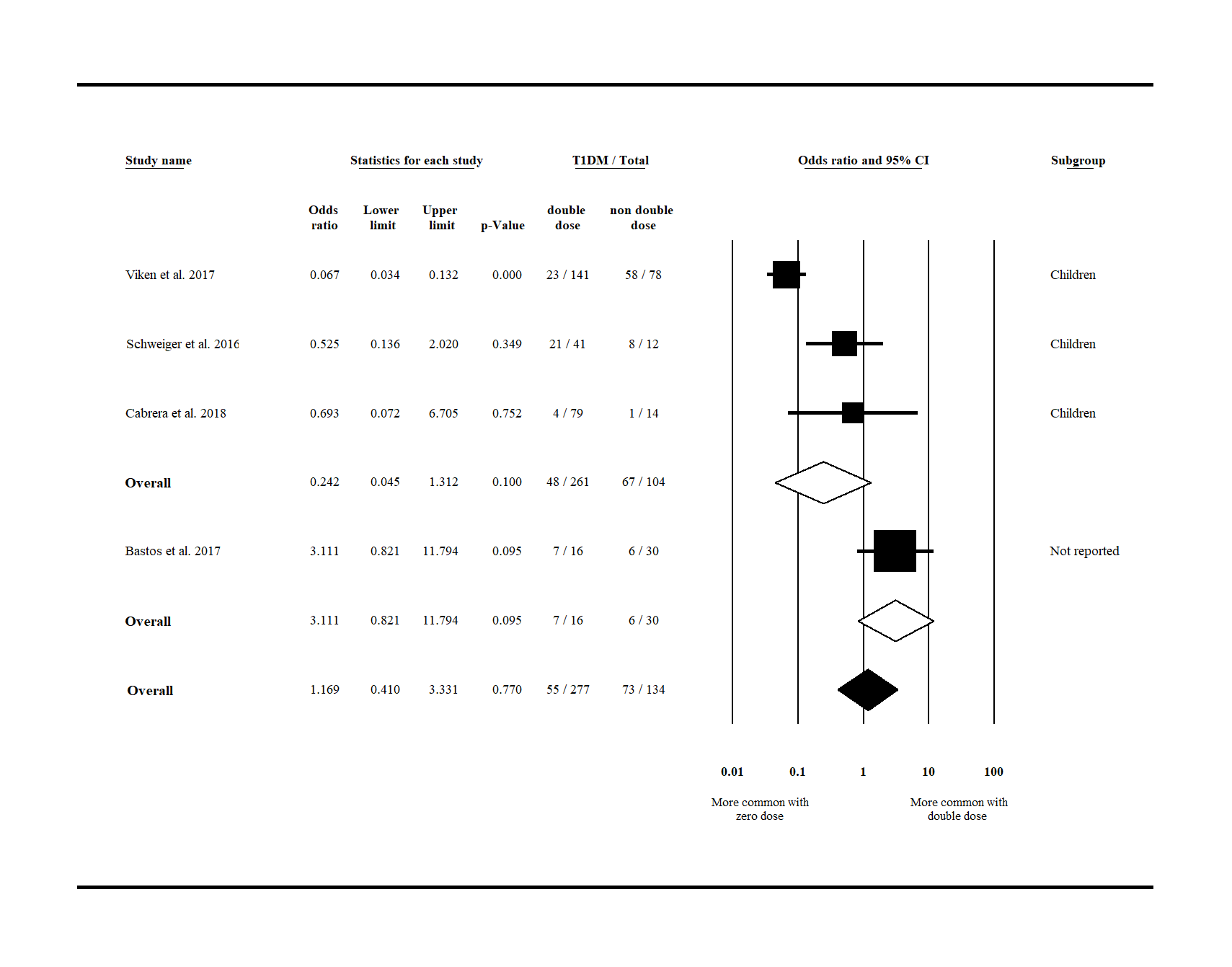
**

Supplement: S8 Fig — CI: confidence interval. (DOCX) [file pone.0212329.s011.docx]
