# Supplementary material for: Classical celiac disease is more frequent with a double dose of HLA-DQB1*02: A systematic review with meta-analysis
Source: PLoS One. 2019 Feb 14;14(2):e0212329. doi: 10.1371/journal.pone.0212329 (PMC6375622; doi:10.1371/journal.pone.0212329)
Supplement: S3 Appendix — (DOCX) [file pone.0212329.s014.docx]

**S3 Appendix**

Age at diagnosis

Atrophic vs. non-atrophic histology

Classical vs. non-classical clinical presentation

Diarrhea vs. no diarrhea

Marsh 3c vs. 3a-b

Type 1 diabetes mellitus
